# Treatment Duration of Each Anti-COVID-19 Agent Used in a Tertiary Hospital in Japan

**DOI:** 10.7759/cureus.100332

**Published:** 2025-12-29

**Authors:** Masafumi Seki, Kazunori Enami

**Affiliations:** 1 Division of Infectious Disease and Infection Control, Saitama Medical University International Medical Center, Hidaka City, JPN; 2 Division of Pharmacy, Saitama Medical University International Medical Center, Hidaka City, JPN

**Keywords:** ensitrelvir, molnupiravir, nirmatrelvir/ritonavir, remdesivir, sars-cov-2

## Abstract

Four anti-COVID-19 agents, nirmatrelvir/ritonavir (Nir/r), ensitrelvir (ESV), molnupiravir (MPV), and remdesivir (RDV), have been used in Japan for adult patients, and their clinical effectiveness, especially treatment duration and reduction of viral antigen titers, was investigated. A total of 114 adult cases received anti-COVID-19 treatment (33 Nir/r, 27 ESV, 24 MPV, and 30 RDV) in our hospital on admission, and the viral antigen titers were sufficiently decreased in all 114 patients. However, the treatment durations differed significantly: 4.09 days with Nir/r, 4.76 days with ESV, 4.74 days with MPV, and 5.08 days with RDV. These data suggest that the anti-viral activity of each anti-COVID-19 agent may differ in clinical use.

## Introduction

COVID-19 has had a huge impact on the world since 2019, and it may still be lethal in patients at high risk, such as elderly men with underlying diseases including malignancies, diabetes mellitus, and obesity [1]. Major guidelines recommend or suggest treatment with antiviral agents for these high-risk patients, although they have mild-to-moderate severity of COVID-19 initially [2-4].

Of the antiviral agents for COVID-19, three oral drugs - nirmatrelvir plus ritonavir (Nir/r; Paxlovid, Pfizer Inc., NY, USA), ensitrelvir (ESV; Shionogi Co., Ltd., Osaka, Japan), and molnupiravir (MPV; Merck, Rahway, NJ, USA) - and one intravenous agent, remdesivir (RDV; Gilead, Foster City, CA, USA), have been used in Japan [2, 5, 6].

Nir/r is administered orally and acts as a protease inhibitor against the 3C-like protease (3CL protease) that affects the viral replication essentially [7]. Nirmatrelvir is combined with ritonavir, which strongly inhibits cytochrome P450 (CYP) 3A4, and co-administration with ritonavir leads to an increase in nirmatrelvir concentrations to a therapeutic range [8]. Therefore, the United States Food and Drug Administration (FDA) approved Nir/r as the treatment agent for mild-to-moderate COVID-19 in adults who have high-risk factors of severe COVID-19 progression in the USA. It was also approved in Japan in February 2022 [8, 9].

Ensitrelvir (ESV; Shionogi Co., Ltd., Osaka, Japan) has been in use in Japan since March 2023, and strong inhibition for SARS-CoV-2 was reported [10, 11]. ESV is also a 3CL protease inhibitor and reduces viral replication effectively. Therefore, treatment with ESV was reported to have clinical improvement in symptomatic COVID-19 patients along with a reduction of SARS-CoV-2 titers [12].

Molnupiravir (MPV) was approved in December 2021 as the first oral anti-viral agent in Japan. MPV is an RNA polymerase inhibitor, and its safety and efficacy is confirmed; it is usually started in patients with at least one risk factor for severe COVID-19 within five days after the onset [13].

RDV is also an RNA polymerase inhibitor and the one and only intravenous agent for SARS-CoV-2; it was found to be associated with a significant reduction in mortality for hospitalized patients, including elderly patients and those with COVID-19 pneumonia [14].

However, the differences in the clinical effectiveness of these four antiviral agents for SARS-CoV-2 are unclear. Therefore, this study aimed to compare treatment duration and changes in antigen titers among hospitalized adults receiving four antiviral agents.

## Materials and methods

Drugs and patients

In this report, the use of Nir/r, ESV, MPV, and RDV at the bedsides, from April 1, 2024 to March 31, 2025, for admitted COVID-19 patients, was retrospectively investigated, examining the differences in treatment duration and changes in viral antigen titers.

Patients under 17 years old were excluded because all four agents were approved for those over 18 years old in Japan. The severity of patients was categorized from mild to moderate, i.e., patients without oxygen therapy to patients who needed oxygen therapy by nasal canula and/or mask [2-4].

Nir/r was used orally - nirmatrelvir 300 mg with ritonavir 100 mg twice daily for five days. ESV was also used orally - 375 mg once on the first day, followed by 125 mg once daily for four more days. Oral MPV was used - 800 mg twice daily for five days. A drip infusion of RDV was administered intravenously - 200 mg once on the first day, followed by 100 mg once daily for four more days. The agents were selected by physicians unbiasedly, but RDV was selected when patients could not accept oral intake.

Ethics

This study and related analyses were approved by the Institutional Review Board of Saitama Medical University International Medical Center on July 6 and March 1, 2023 (#2022-032 and #2022-146), and registered as UMIN000047691. The patients whose specimens and data were analyzed provided written informed consent to have any accompanying images and their case details published. This study was performed according to the Declaration of Helsinki.

Antigen quantification test and determination of isolation duration

The nasal samples were collected by cotton swabs and the SARS-CoV-2 antigen (Ag) test was performed immediately at the clinical laboratory, within 30 minutes after sample collection at the bedside and transported without any unnecessary medium. The SARS-CoV-2 Ag level was measured quantitatively by Cobas SARS-CoV-2 Ag (Roche, Basel, Switzerland).

In our hospital, Ag titers were evaluated on days 3 and 5, and treatment/isolation was ended at day 3 when antigen titers were lower than 100 IU, a level at which the droplet transmission of virus to other patients was considered unlikely, although all antiviral agents generally have a fixed 5-day dosing regimen [15-17]. The reason we measured the Ag level on days 3 and 5, but not day 4, is that all four agents were generally administered by day 5, and RDV was recently recommended 3-day administration for mild patients in both inpatient and outpatient status [2-4].

Statistical analysis

The chi-squared test, ANOVA and the Kruskal-Wallis test were performed for the comparison of continuous variables among four groups. The sample numbers were not huge and might not exclude the confounders. In addition, the samples did not show normal data distributions; therefore, non-parametric analysis was mainly performed, and no additional analysis, such as regression modeling, multivariable or adjusted analysis, was performed. A p-value less than 0.05 was defined as a significant difference. All analyses were performed using StatMate Version VI software (Nihon 3B Scientific, Niigata, Japan), which is the same as Easy R software (Saitama, Japan) and SPSS (IBM Corp., Armonk, NY, USA), available in Japan [18].

## Results

Treatment duration and the viral antigen titers

During this period, a total of 114 patients were treated in our hospital. A total of 33 Nir/r, 27 ESV, 24 MPV, and 30 RDV admitted cases were analyzed (Table 1). All patients were Asian, and clinical backgrounds, including age, male/female ratio, underlying diseases, and vaccination status, were not significantly different.

**Table 1:** Clinical characteristics of patients receiving each anti-COVID-19 agent

		Nir/r (n=33)	ESV (n=27)	MPV (n=24)	RDV (n=30)	p value
Age (Mean, years) (Min-Max)		69.95 (34-86)	76.52 (54-97)	75.58 (49-90)	75.92 (31-92)	0.778
Male/Female		26/7	18/9	16/8	21/9	0.6931
Underlying diseases						0.63
	Heart diseases	9 (27.3%)	11 (40.7%)	5 (20.1%)	5 (16.7%)	0.19
	Malignant tumors	8 (24.2%)	3 (11.1%)	5 (20.1%)	4 (13.3%)	0.5
	Hematological diseases	8 (24.2%)	3 (11.1%)	3 (12.5%)	5 (16.7%)	0.51
	Brain diseases	6 (18.2%)	3 (11.1%)	2 (8.3%)	6 (20.0%)	0.6
	Kidney diseases	1 (3.0%)	3 (11.1%)	3 (12.5%)	4 (13.3%)	0.49
	Rheumatoid diseases	1 (3.0%)	0	2 (8.3%)	1 (3.3%)	0.45
	Neurological diseases	0	1 (3.7%)	1 (4.2%)	2 (6.7%)	0.55
	Diabetes mellitus	0	3 (11.1%)	1 (4.2%)	2 (6.7%)	0.28
	Lung diseases	0	0	2 (8.3%)	1 (3.3%)	0.19
Vaccination (Times)		2	2.1	3.81	2.72	0.055

**Figure 1:**
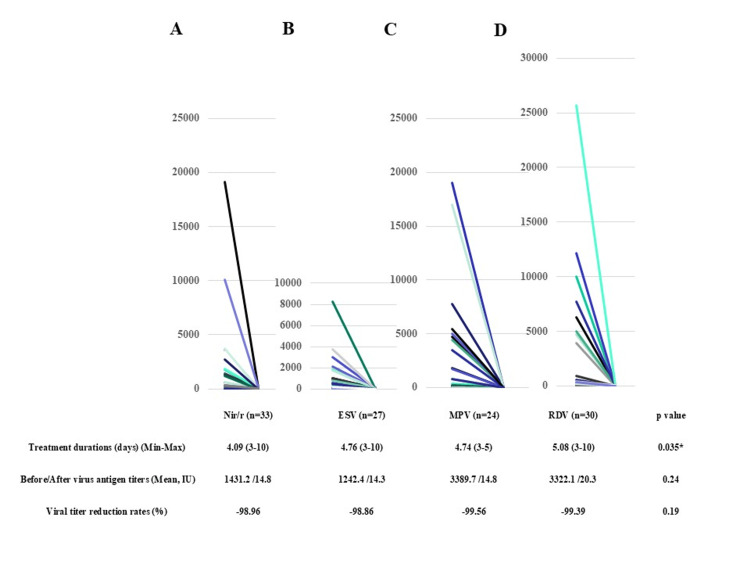
Treatment period duration, change of the antigen titers between before and after treatments, and antigen reduction ratios in patients receiving four different antiviral agents for SARS-CoV-2

Among the patients who received each anti-COVID-19 agent, we found a significantly shorter treatment duration for Nir/r cases (Nir/r 4.09 days, ESV 4.76 days, MPV 4.74 days, and RDV 5.08 days; p=0.035) (Figure 1). The change of antigen titers between before and after treatments was not significantly different, and the antigen reduction ratios were similar among the four agents.

## Discussion

In this report, the results suggested that the four antiviral agents for COVID-19 were generally similar, but Nir/r might be more effective in the real world at the bedside.

Nir/r showed 89% lower risk of progression to severe COVID-19 in the symptomatic patients, compared with placebo, without evident safety concerns in the phase 2-3 double-blind, randomized, controlled trial [8], and the patients treated with Nir/r showed lower risks for death (adjusted risk ratio, 0.29 [CI, 0.12 to 0.71]) and hospitalization (adjusted risk ratio, 0.60 [CI, 0.44 to 0.81]) than placebo in the Omicron period in a population-based cohort study [19]. These data suggest that Nir/r had more clinical effectiveness without changes in the surged virus strain.

In addition, the in vitro antiviral activities of GS-441524, which is a parent nucleoside of RDV; EIDD-1931, which is a parent nucleoside of MPV; and Nir were evaluated against the ancestral SARS-CoV-2 strain and the five variants of concern, including Omicron [20]. All molecules showed equipotent antiviral activity against the ancestral virus and the Alpha, Beta, Gamma, Delta, and Omicron variants. However, RDV and Nir showed about 10 to 100 times lower 50% effective concentrations (EC50) for these SARS-CoV-2 variants compared with MPV, suggesting that RDV and Nir were similar but had more clinical effectiveness than MPV. In addition, Nir was used as Nir/r, which has been boosted by additional ritonavir, and Nir/r appeared to be more effective than RDV. In the present study, Nir/r showed the shortest duration of treatment for COVID-19, and this result may be explained by these basic antiviral experiments.

Furthermore, ESV has a similar mechanism of action as a 3CL-protease like Nir [10], and it has been used for patients with milder disease because the guidelines in Japan recommend that ESV be used for outpatients without high-risk underlying diseases, rather than the moderate-to-severe patients from the clinical study performed mainly during the Omicron period [3, 11, 12]. Therefore, its strong effectiveness for SARS-CoV-2 inhibition might tend to not be clear in real-world practice in Japan. In the present study, ESV was used for patients with relatively lower viral titers than the other three agents. In our previous study, patients who received Nir/r treatment were older and hospitalized more often than patients who received ESV treatment, and the patients who received Nir/r treatment showed more complications with malignant tumors than those who received ESV treatment [6]. These data suggest that Nir/r was administered to the admitted patients who had malignant tumors, and these patients were a small population because most of the admitted patients tended to receive drip infusions of RDV rather than treatments by oral agents when the patients lost good appetite due to the malignant tumors and more severe conditions [5, 16]. In the clinical trials and the real-world studies, Nir/r was administered to patients who had high risks due to severe underlying diseases, although they had mild-to-moderate severity of COVID-19 [8, 9], and it was recommended that Nir/r should be used on those types of patients in both Japan and the USA because it might have strong anti-viral activity for SARS-CoV-2 [2-4].

The study provides real-world comparative data on four antiviral agents used in hospitalized COVID-19 patients, which is clinically relevant. The use of standardized Ag titers and routine hospital monitoring adds consistency. However, this study has a retrospective single-center design. It has limitations because the sample size is not large, which may lead to a generalizability limitation of the results and data. The potential bias in the selection of patients - those who received each antiviral agent - may also be present because more severe patients may tend to receive RDV and Nir/r. In addition, Ag titers were assessed only on days 3 and 5 after treatment initiation. Because treatment duration is discussed with a resolution of less than one day, the lack of measurements on day 4 may limit the precision of estimating when the antigen titer actually crossed the predefined threshold. Further investigations are needed to confirm and establish the relationships between the drugs and patient outcomes.

## Conclusions

The real-world differences of the four antiviral agents for SARS-CoV-2 - Nir/r, ESV, MPV, and RDV - were investigated in this study. The clinical effectiveness of these agents, especially treatment duration, differed, and Nir/r might have the shortest treatment duration due to its strong viral inhibitory activity. More detailed clinical analysis considering the patients’ conditions, including severity and underlying diseases, is needed.
